# Recurrent Bacterial Infections in Cutaneous T-cell Lymphoma

**DOI:** 10.7759/cureus.20912

**Published:** 2022-01-03

**Authors:** Bharadwaj Adithya Sateesh, Yash V Bhagat, Sneha E Thomas, Aseem Sood, Miriam B Michael

**Affiliations:** 1 Medicine, American University of Antigua, St John's, ATG; 2 Internal Medicine, University of Maryland Midtown Campus, Baltimore, USA; 3 Human Genetics, University of Calfornia Los Angeles, Los Angeles, USA

**Keywords:** cellulitis, antibiotics, mycosis fungoides, cancer, infectious disease, immune regulation, staphylococcus aureus, cutaneous t-cell lymphoma

## Abstract

Cutaneous T-cell lymphoma (CTCL) is a dermatologically manifesting immune cell disorder. We present a case of a 76-year-old female with a past medical history of CTCL, presenting with cellulitis of the left foot. After diagnosis of CTCL, the patient was admitted multiple times for treatment of cutaneous and soft-tissue infections with methicillin-resistant *Staphylococcus aureus*. Her recurrent infection with* S. aureus* had led to treatment for sepsis and a below-knee amputation on the right during prior hospitalizations. On this admission, the patient was treated with intravenous vancomycin and cefepime as in-patient and oral linezolid as out-patient. Recent articles show that patients with CTCL have an increased tendency to harbor *S. aureus*, which leads to recurrent infections. Additionally, evidence suggests that *S. aureus *toxins aid the progression of CTCL by helping the cancer to escape immune regulation. Our patient demonstrates this unique relationship between CTCL and *S. aureus, *and moreover, we make a case that *S. aureus* infection in CTCL, as compared to that in other dermatitis, should be better managed to not exacerbate the disease.

## Introduction

Cutaneous lymphomas may originate from T, B, or natural killer cells. Mycosis fungoides (MF) is a type of cutaneous T-cell lymphoma (CTCL) that originates from epidermal T cells, and it expresses the T-cell receptor (TCR) and CD4+ immune cell markers. Although the etiology of the disease is unclear, epigenetic anomalies (DNA methylation, microRNAs, histone modifications), environmental predispositions (pollution, chemical exposure, pesticides, detergents), and infectious agents such as the human T-lymphotropic virus (HTLV) type 1 have been hypothesized as potential causes of the disease. Sezary syndrome is a leukemic form of CTCL where large lymphocytes are found in the peripheral blood with “cerebriform” nuclei [1]. Skin biopsy with routine monitoring of histopathology is the best way to establish a diagnosis of CTCL [2]. Skin biopsies demonstrate mononuclear cells with cerebriform nuclei infiltrating the upper dermis or forming intraepidermal aggregates known as Pautrier microabscesses. Immunophenotype and TCR gene rearrangement studies can be used to support or confirm results of the routine histology in patients whose clinical presentation is strongly suggestive of MF. Patients from certain geographic regions such as the Caribbean, West Africa, and Japan need to have HTLV serology performed as well. Unlike Sezary syndrome, which manifests with dermal pruritus, lymphadenopathy, and atypical circulating lymphocytes, MF does not present with circulating lymphocytes and is thus easily confused for dermatological rashes, such as atopic dermatitis or psoriasis [3]. Cultures for microbes that infect CTCL patients have demonstrated associations with *Staphylococcus aureus*, retroviruses, and herpesviruses [4]. Recent studies have hypothesized that patients with CTCL not only harbor an increased number of these microbes but that the microbes in turn aid in the progression of CTCL. Our case report is an example of this increased microbial burden faced by patients with CTCL. 

## Case presentation

**Figure 1:**
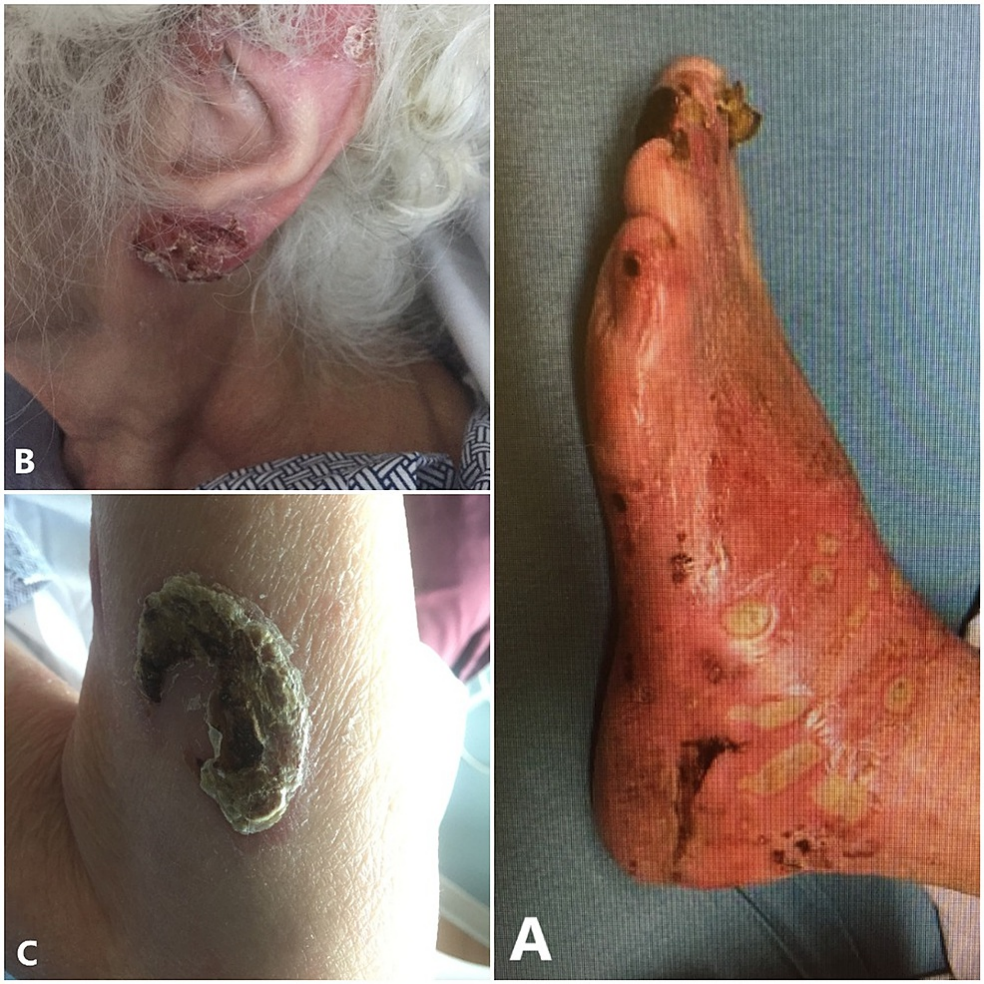
Ulcerations found on the patient's foot (A), scabbing lesion found on the ear (B), and plaque on the forearm (C). All photographs were taken with the permission of the patient.

A 76-year-old female with a past medical history of CTCL diagnosed in 2016, peripheral artery disease, below-knee amputation of the right leg, and type 2 diabetes mellitus complicated by chronic urinary incontinence presented to the emergency department with complaints of foot ulcers. The patient has diabetic neuropathy and foot numbness. She admitted to wearing the same sock for a week and that it was usually soaked in urine due to her incontinence. She removed the sock after she developed pain and found multiple ulcers on her left foot with erythematous base (Figure 1A). She also endorsed painful rashes and ulcers in her upper body, inguinal area, torso, and palms . She was found on admission to have leukocytosis with a white blood cell count of 11.5 x 10^3^cells/μL and radiological testing with X-ray showed no signs of osseous erosion associated with osteomyelitis. She was started on empiric vancomycin and cefepime and was given a tetanus booster. 

On examination the left foot was erythematous up to the ankle with multiple ulcerations, and hard scabbing lesions were found over the left ear and nares (Figure 1A, 1B). A large plaque was noticed over the left forearm (Figure 1C). The infected area was warm to the touch but no crepitus was felt. She had 2+ dorsalis pedis and popliteal pulses. On day 3 of treatment, her leukocytosis had resolved and she was transitioned from IV vancomycin and cefepime to oral linezolid 600 mg twice a day for five days.

The patient initially presented with lesions in 2014 that were diagnosed as psoriaform dermatitis and underwent subsequent treatment with UV exposure. On follow-up two years later in 2016, a repeat biopsy of these same lesions revealed an infiltrate of atypical lymphocytes in the upper dermis and epidermis with absence of spongiosis. On pathology, the lesion was found to be CD30+; this was indicative of large cell transformation of MF. Initial treatment for her malignancy involved brentuximab vedotin, which was discontinued due to severe neuropathy. She was also treated with radiation therapy and narrowband ultraviolet therapy. In December of 2019, she developed necrotizing fasciitis from right lower extremity cellulitis. She had bacteremia with blood cultures positive for methicillin-resistant *S. aureus*. She quickly developed sepsis and had to undergo a right lower limb below-knee guillotine amputation. A combination of decreased mobility and COVID lock-down caused her to become inconsistent with her treatment for the last two years. A positron emission tomography scan six months ago showed increased metabolic activity at the right thigh, right flank, left buttock, and left periareolar region. Treatment with eight cycles of brentuximab vedotin was resumed. 

## Discussion

A 76-year-old female with a past medical history of CTCL presented with pain in her left foot that was concerning for cellulitis. The patient has also had multiple previous visits in the past for skin infections. There is a strong association of recurrent infections in patients with CTCL . These bacterial infections are a major problem in patients due to the compromised skin barrier and progressive immunodeficiency. The immunodeficiency in CTCL is multifactorial. The primary cause is believed to be cytokine-dependent as there is a bias toward the cytokines produced by TH2 cells over TH1, which obstructs an effective cellular response. Further, there is increased expression of programmed death protein 1 (PD1) and its corresponding ligand (PD-L1) and this interaction has been associated with immune cell evasion in CTCL. PD-L1 has been hypothesized to activate the Janus kinase (JAK)-signal transducer and activator of transcription (STAT) pathway, which induces the secretion of potent immunosuppressant cytokines interleukin 10 (IL-10) and transforming growth factor B (TGF-B) [5]. 

A literature review performed on PubMed with the keywords “Cutaneous T cell lymphoma" and "staphylococcal infections” yielded 36 results. Inclusion criteria were applied, and only studies written in English and conducted in humans in the last 10 years (articles published between 2011 and 2021) were included, yielding 11 results. Articles that were relevant to our topic and research question (the role of *S. aureus* in CTCL), peer reviewed, full texts, and including the types classical article and clinical studies were included. Duplicates of articles, articles not written in English, books and book chapters, letters to the editor, case reports, opinionated articles, editorials, or letters, and in vitro or animal studies were excluded from the literature review. Articles that were strictly abstracts or poster presentations were excluded. Narrative and systematic reviews, meta-analyses, and other literature reviews were excluded, giving two articles to be reviewed [5,6]. We acknowledge that despite our best efforts, some relevant articles may have been missed.

*S. aureus* is a major cause of morbidity in patients with CTCL causing chronic and recurrent skin and systemic infections ranging from cellulitis to sepsis [5]. Staphylococcal enterotoxins contribute to disease progression in CTCL, namely, the alpha-toxin comprehensively blocks the cytotoxic killing of CTCL cells thus aiding in tumor evasion from programmed cell death [5,7].* S. aureus* enterotoxins also induce the expression of oncogenic microRNA mIR-155 in malignant T cells [8]. MF lesions harboring *S. aureus* express Y-phosphorylated STAT5, and display enhanced miR-155 expression when compared to normal healthy skin [8,9]. A particularly important and interesting association seen here is that when treated with antibiotics, there is decreased Y-phosphorylated STAT5 and miR-155 expression in lesioned skin in patients [8]. A study in which patients were tested for the association of *S. aureus w*ith CTCL showed that 76% of the patients were positive for enterotoxin-producing* S. aureus,* thus indicating that paying heed to these toxigenic strains can potentially improve the outcomes in patients with CTCL [9].

*S. aureus* is the most common harboring organism in any form of dermatitis including CTCL [10]. Treatment of these infections usually involves Gram-positive coverage with agents like vancomycin or linezolid. Since our patient was diabetic, cefepime was added in order to cover for pseudomonas. Prompt treatment with antibiotics is indeed warranted as patients with CTCL usually undergo worse courses with infections in comparison to the general population or patients with other forms of dermatitis [6]. However, we feel that long-term antibiotic therapy is not warranted and should be reserved for treatment of overt infections. Interestingly, a study of atopic dermatitis treatment with long-term antibiotics revealed recolonization with *S. aureus* by four weeks after stopping the antibiotics, rendering the benefits of long-term antibiotic use moot [11]. Moreover, we posit that the use of antibiotics as prophylaxis may result in the creation of multi-drug-resistant microbes. We think that moving forward, in patients with CTCL, treatment of symptomatic infections along with strict hygiene is highly important to prevent recurrent superinfections by lesions harboring *S. aureus*. Recommended methods include hand-washing and clean living environment [12,13]. Decolonization in the out-patient setting is also another way and can be done either nasally with mupirocin or topical body decolonization with either daily washes of chlorhexidine gluconate or dilute bleach baths [13,14]. However, there is no optimal decolonization technique nor is there any significant data to demonstrate the efficacy of these methods.

## Conclusions

A case of CTCL was presented where the patient had multiple cutaneous infections, which were *S. aureus* associated. The cutaneous lesions harbor this organism and the natural toxins produced by this organism accelerate the disease progression. This also commenced a discussion on the mechanism by which it occurs and whether treating the microbe slows the progression of the lymphoma. Preventative methods including decolonization, proper hygiene, and sanitization of these patients are essential in the prevention of infections, as if they usually get infected the course is tortuous. This case warrants additional quantitative research into whether treatment slows down the progression and into the exact immune-evading mechanisms utilized by *S. aureus*.

## References

[1] Wilcox RA (2017). Cutaneous T-cell lymphoma: 2017 update on diagnosis, risk-stratification, and management. Am J Hematol.

[2] Fung MA, Murphy MJ, Hoss DM, Grant-Kels JM (2002). Practical evaluation and management of cutaneous lymphoma. J Am Acad Dermatol.

[3] Vaidya T, Badri T (2021). Mycosis fungoides. StatPearls [Internet].

[4] Mirvish ED, Pomerantz RG, Geskin LJ (2011). Infectious agents in cutaneous T-cell lymphoma. J Am Acad Dermatol.

[5] Willerslev-Olsen A, Krejsgaard T, Lindahl LM (2013). Bacterial toxins fuel disease progression in cutaneous T-cell lymphoma. Toxins (Basel).

[6] Lebas E, Arrese JE, Nikkels AF (2016). Risk factors for skin infections in mycosis fungoides. Dermatology.

[7] Blümel E, Munir Ahmad S, Nastasi C (2020). Staphylococcus aureus alpha-toxin inhibits CD8+ T cell-mediated killing of cancer cells in cutaneous T-cell lymphoma. Oncoimmunology.

[8] Willerslev-Olsen A, Gjerdrum LM, Lindahl LM (2021). Staphylococcus aureus induces signal transducer and activator of transcription 5‒dependent miR-155 expression in cutaneous T-cell lymphoma. J Invest Dermatol.

[9] Jackow CM, Cather JC, Hearne V, Asano AT, Musser JM, Duvic M (1997). Association of erythrodermic cutaneous T-cell lymphoma, superantigen-positive Staphylococcus aureus, and oligoclonal T-cell receptor V beta gene expansion. Blood.

[10] Nakatsuji T, Chen TH, Narala S (2017). Antimicrobials from human skin commensal bacteria protect against Staphylococcus aureus and are deficient in atopic dermatitis. Sci Transl Med.

[11] Friedman BC, Goldman RD (2011). Anti-staphylococcal treatment in dermatitis. Can Fam Physician.

[12] Pittet D, Hugonnet S, Harbarth S, Mourouga P, Sauvan V, Touveneau S, Perneger TV (2000). Effectiveness of a hospital-wide programme to improve compliance with hand hygiene. Infection Control Programme. Lancet.

[13] Calfee DP, Salgado CD, Milstone AM (2014). Strategies to prevent methicillin-resistant Staphylococcus aureus transmission and infection in acute care hospitals: 2014 update. Infect Control Hosp Epidemiol.

[14] Liu C, Bayer A, Cosgrove SE (2011). Clinical practice guidelines by the Infectious Diseases Society of America for the treatment of methicillin-resistant Staphylococcus aureus infections in adults and children. Clin Infect Dis.

